# ESTELA‐Study: Long‐Term Effectiveness and Safety of Anti‐Calcitonin Gene‐Related Peptide Monoclonal Antibodies in Real‐World Clinical Practice

**DOI:** 10.1002/brb3.71560

**Published:** 2026-06-25

**Authors:** Alba Somovilla, Iris Fernández‐Lázaro, Josué Pagán, Daniel Saiz, Javier Díaz‐De‐Terán, Leonardo Portocarrero Sánchez, Germán Latorre, Carlos Calle De Miguel, Nuria González‐García, María‐Luz Cuadrado, Jesús Porta‐Etessam, Javier Casas‐Limón, David Garcia‐Azorin, Ángel Guerrero‐Peral, Yésica González‐Osorio, Alicia Gonzalez‐Martinez, Guillermo Martín Ávila, Rodrigo Terrero Carpio, Jaime Rodríguez‐Vico, Alex Jaimes, Andrea Gómez García, Cristina Trevino‐Peinado, Margarita Sanchez‐Del‐Rio, Alberto Lozano Ros, Antonio Sánchez‐Soblechero, Sarai Urtiaga Valle, Marta González‐Salaices, Elena Riva, Ana Gago‐Veiga

**Affiliations:** ^1^ Headache Unit, Department of Neurology, Health Research Institute La Princesa University Hospital Madrid Spain; ^2^ Electronic Engineering Department Universidad Politécnica de Madrid Madrid Spain; ^3^ CCS: Center for Computational Simulation Universidad Politécnica de Madrid Madrid Spain; ^4^ Headache Unit, Department of Neurology, Health Research Institute (IdiPAZ) La Paz University Hospital Madrid Spain; ^5^ Headache Unit, Department of Neurology Fuenlabrada University Hospital Madrid Spain; ^6^ Headache Unit, Department of Neurology, Health Research Institute Clínico San Carlos Hospital Madrid Spain; ^7^ Department of Medicine School of Medicine Universidad Complutense de Madrid Madrid Spain; ^8^ Headache Unit, Department of Neurology Fundación Jiménez Díaz University Hospital Madrid Spain; ^9^ Headache Unit, Department of Neurology Fundación Alcorcón University Hospital Madrid Spain; ^10^ Headache Unit, Department of Neurology Río Hortega Hospital Valladolid Spain; ^11^ Department of Medicine University of Valladolid (UVA) Valladolid Spain; ^12^ Headache Unit, Department of Neurology Clínico De Valladolid University Hospital Valladolid Spain; ^13^ Valladolid Biosanitary Research Institute (IBIoVALL) Valladolid Spain; ^14^ Headache Unit, Department of Neurology Getafe University Hospital Madrid Spain; ^15^ Headache Unit, Department of Neurology Severo Ochoa University Hospital, Leganés Madrid Spain; ^16^ Faculty of Biomedical and Health Sciences Universidad Alfonso X el Sabio (UAX) Madrid Spain; ^17^ Headache Unit, Department of Neurology Clínica Universidad de Navarra Madrid Spain; ^18^ Headache Unit, Department of Neurology Gregorio Marañón University Hospital Madrid Spain; ^19^ Headache Unit, Department of Neurology Torrejón University Hospital Madrid Spain; ^20^ Pediatric Neurology Area Neurology Service of the Ruber International Hospital Madrid Spain; ^21^ Headache Unit, Department of Neurology Ruber Internacional Hospital Madrid Spain; ^22^ Department of Medicine Universidad Autónoma de Madrid Madrid Spain

**Keywords:** Anti‐CGRP monoclonal antibody, effectiveness, headache, long‐term, migraine, real‐world evidence, safety, treatment outcomes

## Abstract

**Background:**

Anti‐CGRP antibodies are effective and safe in real‐world migraine management, but guidelines recommend discontinuation after 12–18 months due to limited long‐term data and remaining uncertainties regarding optimal treatment duration and sustained safety, highlighting the need for large‐scale long‐term real‐world evidence. This study evaluated their safety and effectiveness in patients treated for ≥2 years.

**Methods:**

This multicenter retrospective study included patients from 13 headache units who received the same anti‐CGRP antibody for ≥24 months, excluding discontinuation periods. Baseline characteristics, monthly headache days (MHD), monthly migraine days (MMD), and adverse events (AEs) were recorded at baseline, 6 months, 1, 2, 3, and 4 years. Descriptive statistics were used to summarize clinical characteristics, and appropriate parametric or non‐parametric tests were applied for group comparisons. Multivariate analyses were performed to explore associations between baseline variables and long‐term treatment response.

**Results:**

A total of 454 patients (91% female, mean age 48) were analyzed, with follow‐up at 2 years (*n* = 454), 3 years (*n* = 135), and 4 years (*n* = 17). Treatments included erenumab (39%), galcanezumab (34%), and fremanezumab (27%). Fifty‐seven percent maintained continuous therapy, while 43% restarted after discontinuation. Sustained reductions in MHD and MMD were observed at 2, 3, and 4 years (MHD from 20 to 6, 6, 5/MMD from 14 to 4, 4, and 2). Medication overuse decreased from 78% to 13%, 20%, and 18%. Loss of effectiveness occurred in 4.2% after 2 years. AEs appeared in <20%, mostly mild (>80%), leading to discontinuation in 0.4%. Multivariate analysis showed that shorter disease duration prior to anti‐CGRP initiation, earlier anti‐CGRP initiation, and greater MHD/MMD reduction at 6 months were associated with better long‐term outcomes.

**Conclusions:**

Anti‐CGRP mAbs demonstrate sustained long‐term safety and effectiveness, with consistent reduction in headache and migraine days and lower medication overuse. Early initiation and greater initial improvement predict better long‐term outcomes. Findings support extending therapy beyond 12–18 months, supporting optimization of clinical protocols.

**Abbreviations**
AE: Adverse Effect
AMSM: Acute Migraine‐Specific Medication
CGRP: Calcitonin Gene‐Related Peptide
CM: Chronic Migraine
HFEM: High‐Frequency Episodic Migraine
HIT‐6: Headache Impact Test‐6
IQR: Interquartile Range
LFEM: Low‐Frequency Episodic Migraine
mAbs: Monoclonal Antibodies
MHD: Monthly Headache Days
MMD: Monthly Migraine Days
MO: Medication Overuse
OLE: Open‐Label Extension
PROs: Patient‐Reported Outcomes
QoL: Quality of Life
SD: Standard Deviation

## 1 Introduction

Migraine is a prevalent, chronic, and debilitating neurological disorder that significantly impairs quality of life and represents a leading cause of long‐term disability (GBD 2016). It affects approximately one billion individuals worldwide each year, predominantly middle‐aged individuals of working age (Kung et al. 2023). Migraine is also more prevalent in women than in men, with global age‐standardized prevalence estimates of approximately 19% and 10%, respectively (Kung et al. 2023).

Based on the available literature, it is estimated that nearly half of the patients would fulfill the criteria to start preventive treatment (Lipton et al. 2007). Recent therapeutic advancements in migraine treatment have led to the development of monoclonal antibodies (mAbs) targeting the calcitonin gene‐related peptide (CGRP) pathway, which has been reported to play a key role in migraine pathophysiology (Durham et al. 2004). Over the past decade, the introduction of anti‐CGRP mAbs as a preventive treatment approach has markedly changed the treatment landscape. Currently, four anti‐CGRP mAbs—galcanezumab, erenumab, fremanezumab, and eptinezumab—demonstrated favorable efficacy and tolerability profiles in multiple 12‐ to 24‐week double‐blind trials (Dodick 2019; Silvestro et al. 2023; Russo and Hay 2023; Santos‐Lasaosa et al. 2022; Serra López‐Matencio et al. 2022; Schiano di Cola et al. 2023; Khalili et al. 2026; Shakir et al. 2026), and they are available for clinical use. Beyond randomized trial durations, real‐world observational studies have reported effectiveness and safety outcomes at follow‐up periods of approximately 48–52 weeks (Barbanti et al. 2024; Vernieri et al. 2024; Fernández‐Bravo‐Rodrigo et al. 2024; Fernández‐Bravo‐Rodrigo et al. 2026). However, data beyond the first year of treatment in routine clinical practice remain limited.

Current European guidelines recommend a period of treatment with anti‐CGRP mAbs between 12 and 18 months, followed by a period of treatment discontinuation (Sacco et al. 2020; Sacco et al. 2022).

This recommendation is primarily based on the lack of long‐term real‐world data rather than on robust evidence regarding effectiveness or safety to support treatment discontinuation. Most available evidence stems from pivotal trials and long‐term open‐label extension (OLE) studies, which have demonstrated sustained efficacy for up to 5 years (Ashina et al. 2021; Pozo‐Rosich et al. 2022; Friedman and Cohen 2020; Kudrow et al. 2021).

However, these studies involve highly selected populations with strict inclusion criteria, limiting their generalizability to routine clinical practice.

Emerging real‐world observational data suggests that many patients experience clinical worsening after treatment discontinuation (Schiano di Cola et al. 2023; Latorre González et al. 2024), raising concerns about the appropriateness of a fixed‐duration treatment strategy. Furthermore, although most patients regain response upon treatment reinitiation, temporary withdrawal may lead to clinical deterioration and negatively impact patients’ quality of life (Romero del Rincón et al. 2024).

These findings highlight the need for large‐scale, multicenter real‐world studies to evaluate the long‐term safety and effectiveness of anti‐CGRP mAbs.

The objective of this study was to evaluate the durability of effectiveness and safety of anti‐CGRP mAbs on headache outcomes and treatment tolerability in adult patients with migraine undergoing prolonged treatment in a real‐world setting, with follow‐up extended up to 48 months.

## 2 Methods

The ESTELA study is a multicenter, observational, retrospective analysis conducted in 13 Headache Units in Spain, which collects clinical data on patients who received treatment with anti‐CGRP mAbs over the period from 2019 to 2024.

### 2.1 Eligibility Criteria

Patients diagnosed with migraine according to the International Classification of Headache Disorders, third edition, who had completed at least 2 years of treatment with the same anti‐CGRP mAb (erenumab, galcanezumab, or fremanezumab) were included. No patients treated with eptinezumab were included, as none had completed at least two years of treatment at the time of data collection. Anti‐CGRP mAbs prescription guidelines (Agencia Española de Medicamentos y Productos Sanitarios 2019; Agencia Española de Medicamentos y Productos Sanitarios 2019; Agencia Española de Medicamentos y Productos Sanitarios 2020) and the European Headache Federation definition of resistant migraine (Sacco et al. 2020; Sacco et al. 2022) were followed. Inclusion criteria permitted continuous or intermittently interrupted treatment, provided that patients completed two full years of active therapy, excluding off‐treatment periods.

In turn, patients who switched to a different anti‐CGRP mAb were excluded to allow for the evaluation of long‐term safety with the same agent, as well as those with less than two years of follow‐up.

### 2.2 Study Variables and Outcome Measures

Long‐term treatment was defined in this study as continuous exposure to anti‐CGRP mAb therapy for ≥24 months, with extended follow‐up analyses up to 48 months to evaluate sustained effectiveness and safety in patients with the longest treatment duration.

Demographic data and clinical‐therapeutic features were systematically recorded through the electronic medical records at baseline and 6, 12, 24, 36, and 48 months after anti‐CGRP mAb onset.

Effectiveness variables included the change from baseline in monthly headache days (MHD), in monthly migraine days (MMD), and in monthly acute migraine‐specific medication (AMSM).

Safety and tolerability were assessed through the documentation of adverse events across different time periods. Quality of life and disability variables were described using the questionnaire Headache Impact Test 6 (HIT‐6) (Kosinski et al. 2003).

### 2.3 Statistical Analysis

#### 2.3.1 Statistical Tests Applied

Statistical analyses were conducted to evaluate changes from baseline and the persistence of therapeutic effects over time. Descriptive statistics summarized demographic and clinical characteristics. Between‐group comparisons were performed using Fisher's exact or Pearson's chi‐square test for categorical variables and the Wilcoxon rank‐sum test for continuous variables, with a significance threshold of *p* < 0.05. Data distribution was assessed with standard normality tests; ANOVA or Kruskal–Wallis was applied accordingly.

Multivariate analyses were performed to explore associations between baseline clinical characteristics and long‐term treatment response (≥24 months). Predictive modeling included logistic regression and selected machine learning methods (random forests and gradient boosting), trained on baseline clinical data. Results are presented as mean ± SD, median [IQR], or frequency (%), as appropriate.

#### 2.3.2 Subgroup Definition for Multivariate Analysis

We conducted a multivariate analysis to identify variables associated with different patterns of response to anti‐CGRP mAbs at 24 months, based on three key aspects:

- **Magnitude of response**: <25%, 25–50%, or >50%.
- **Time to achieve ≥50% improvement in response**: before 12 months, by 24 months, or not achieved by either 12 or 24 months.
- **Duration of response**: sustained (>50% at 24 months) or non‐sustained (<50% at 24 months)

#### 2.3.3 Subgroups at 24‐months Analysis

Patients were categorized into five subgroups based on their response to treatment during the first two years in comparison with baseline (Table 1a), in line with the methodology previously described in the literature (Torres‐Ferrus et al. 2023).

- **A‐0. Sustained‐responders**: Patients with clinically significant improvement (≥50%) maintained at both 12 and 24 months—ideal group, indicating sustained effectiveness.
- **A‐1. Non‐sustained responders**: Patients with ≥50% improvement at 12 months that declines to 25%–50% by 24 months, indicating partial loss of long‐term effectiveness.
- **A‐2. Ultra‐Late responders**: Patients without marked initial response who achieve a ≥50% improvement at 24 months, reflecting delayed therapeutic effect with subsequent sustained effectiveness.
- **A‐3. Limited responders**: Patients with a moderate response (25%–50%) from the outset, maintained at least through 12 months, retaining a clinically relevant effect.
- **A‐4. Low responders**: Patients with < 25% improvement at both time points are considered low‐responders.

**TABLE 1:** **Classification of patients according to response to anti‐CGRP treatment** based on percentage reduction in monthly headache and migraine days at different time points.

a)	M12	M24
**A‐0. Sustained‐responders**	**≥50%**	**≥50%**
**A‐1. Non‐sustained responders**	**≥50%**	**25%‐49%**
**A‐2. Ultra‐Late responders**	**25%‐49%**	**≥50%**
**A‐3. Limited responders**	**25%‐49%**	**25%‐49%**
***A‐4. Low responders***	**<25%**	**<25%**

b)	M24	M36
**B‐0**	**≥50%**	**≥50%**
**B‐1**	**≥50%**	**<50%**
**B‐2**	**<50%**	**≥50%**
**B‐3**	**<50%**	**<50%**

#### 2.3.4 Subgroups at 36‐months Analysis

Patients were categorized into four subgroups based on their response to treatment during the third year of treatment (Table 1b):

- **B‐0. Ultra‐sustained responders**: Patients achieving ≥50% improvement at 24 months and maintaining it at 36 months.
- **B‐1. Late‐sustained responders**: Patients whose response was ≥50% at 24 months but declined by 36 months.
- **B‐2. Ultra‐delayed responders**: Patients who did not reach ≥50% improvement at 24 months but achieved it by 36 months.
- **B‐3. Maintained‐limited responders**: Patients with low response (<50%) at both 24 and 36 months.

### 2.4 Missing Data

Variables with more than 20% missing data were excluded. Missing values were assessed by evaluating the proportion of missing data per variable. Variables were stratified according to their type (integer numerical, continuous numerical, and categorical). Numerical variables were imputed using a k‐nearest neighbors (KNN) imputation approach, while categorical variables were imputed using an iterative imputation method. Data were subsequently normalized when required for modeling.

## 3 Results

### 3.1 Clinical and Demographic Features

**FIGURE 1:**
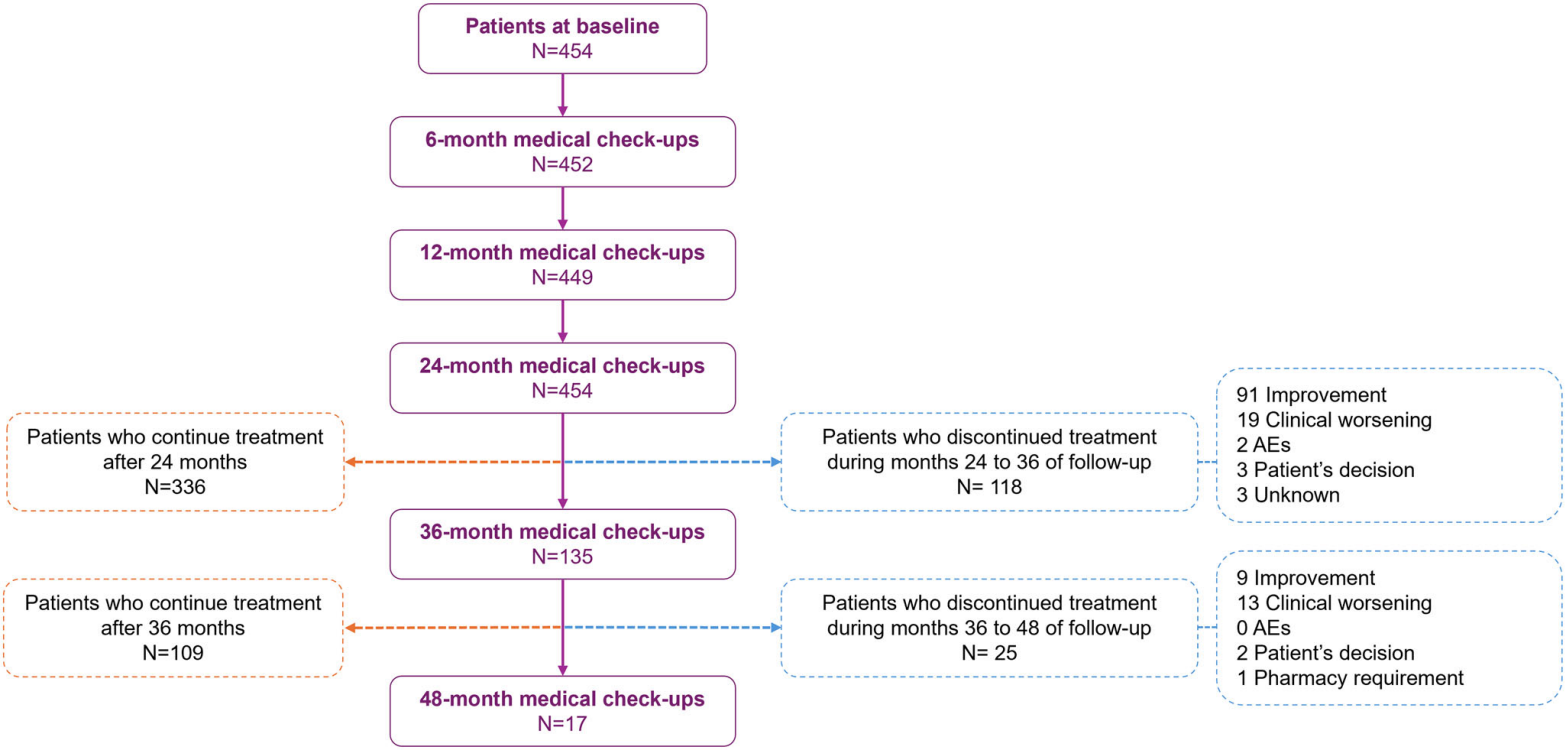
**Flowchart of patients**. The study included patients who had received treatment with anti‐CGRP monoclonal antibodies for a minimum duration of two years. The treatment period ranged from 2019 to 2024. *Only 17 patients have reached the 4‐year follow‐up under treatment.

A total of 454 patients were included in the study. All of them completed 2 years of treatment (24 months) with anti‐CGRP mAbs and attended the 2‐year clinical visit. After this visit, 118 patients discontinued treatment (91 due to clinical improvement, 19 due to worsening, 2 due to adverse events, 3 by patient decision, and 3 for unknown reasons). The remaining patients continued treatment; however, due to the longitudinal nature of the cohort, 336 had not yet reached 36 months of treatment at the data cutoff. Among the 135 patients who completed 3 years of treatment (36 months), 25 discontinued therapy (9 due to clinical improvement, 13 due to worsening, 2 by patient decision, and 1 due to pharmacy requirements). The remaining patients continued therapy; however, only 17 had completed 4 years of treatment (48 months), while the other 93 had not yet reached this time point at the data cutoff. Details are shown in the flowchart (Figure 1). It should be noted that the progressive reduction in sample size at 36 and 48 months reflects the retrospective design and the staggered entry of patients across centers, meaning that at the data cutoff many had not yet reached these follow‐up milestones, rather than indicating a high rate of attrition.

The study cohort consisted of 454 patients (Table 2), mostly female (413/454, 91%), with a median age of 48 years (IQR: 42–55) at the initiation of anti‐CGRP mAbs. Chronic migraine (CM) was the most prevalent diagnosis, affecting 72% of patients, followed by high‐frequency episodic migraine (HFEM), observed in 27% of cases. A diagnosis of low‐frequency episodic migraine (LFEM) was reported in only 2 patients (0.4%), both of whom initiated monoclonal antibody therapy as an elective treatment in private practice and was continued thereafter.

**TABLE 2:** ***Demographic and clinical characteristics***. *Note: Continuous data are represented as median (interquartile range) and categorical data as n (%)*.

Characteristics	Patients (N= 456)
**Sex**	
**Male**	41 (9.0%)
**Female**	415 (91%)
**Diagnosis**	
**LFEM**	2 (0.4%)
**HFEM**	125 (27%)
**CM**	328 (72%)
**Aura**	121 (26.5%)
**Age at onset**	29 (19, 36)
**Age at chronification**	35 (28, 43)
**Number of preventive treatments**	7.0 (5.0, 9.0)
**Age at antibody onset**	48 (42, 55)
**Months on treatment**	31 (26, 38)
**Intermediate discontinuation**	197 (43%)
**Worse after 24 months**	31 (6.8%)

In relation to the migraine features, the median age of migraine chronification was 35 (IQR: 28–43) years. We observed a median of 7 (IQR: 5–9) previously failed prophylactic treatments. Among the prescribed anti‐CGRP mAbs, erenumab accounted for 39% of treatments, galcanezumab for 34% and fremanezumab for 27%. Continuous treatment was maintained by 57% of patients, whereas 43% underwent treatment reintroduction after previous intermittent cessation, with a median discontinuation period of 5 months (IQR: 3–8).

#### 3.1.1 Long‐Term Effectiveness

In terms of treatment outcomes, a sustained reduction in both monthly headache days (MHD) and monthly migraine days (MMD) was observed at 2 and 3 years of anti‐CGRP mAbs therapy, compared to baseline. A similar reduction was also observed at 4 years of treatment; however, these results should be interpreted with caution due to the small sample size at this time point

**FIGURE 2:**
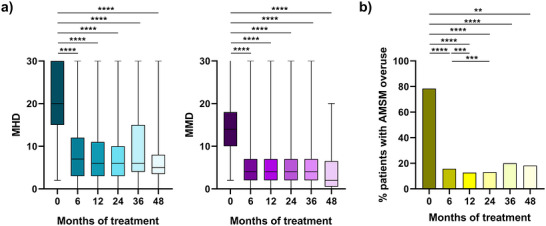
**(a)** Long‐term effectiveness during 3 years of anti‐CGRP mAb therapy. Representation of monthly headache days (MHD) and monthly migraine days (MMD) at baseline, 6, 12, 24, 36, and 48 months of treatment. Box plots represent the median and interquartile range (IQR), with individual data points shown as dots. Error bars represent minimum and maximum values. **(b)** Evolution of acute migraine‐specific medication (AMSM) overuse prevalence. *Note*: ****p < 0.0001, ***p < 0.001, **p < 0.05, and *p < 0.01.

Focusing on the second year of treatment, MHD decreased from a median of 20 days (IQR: 15–30) to 6 days (IQR: 3–10), while MMD dropped from 14 days (IQR: 10–18) to 4 days (IQR: 2–7). These results remained stable at the 3‐year follow‐up. At 4 years (48 months), patients who continued treatment showed a further sustained reduction, reaching 5 MHD (IQR: 3.5‐8) and 2 MMD (IQR: 0.5‐6.5) (Figure 2a).

Notably, acute migraine‐specific medication (AMSM) overuse decreased significantly from 78.4% at baseline to 13%, 20% and 18% at two, three and four years of treatment, respectively (Figure 2b).

### 3.2 Long‐term Safety and Tolerability

**FIGURE 3:**
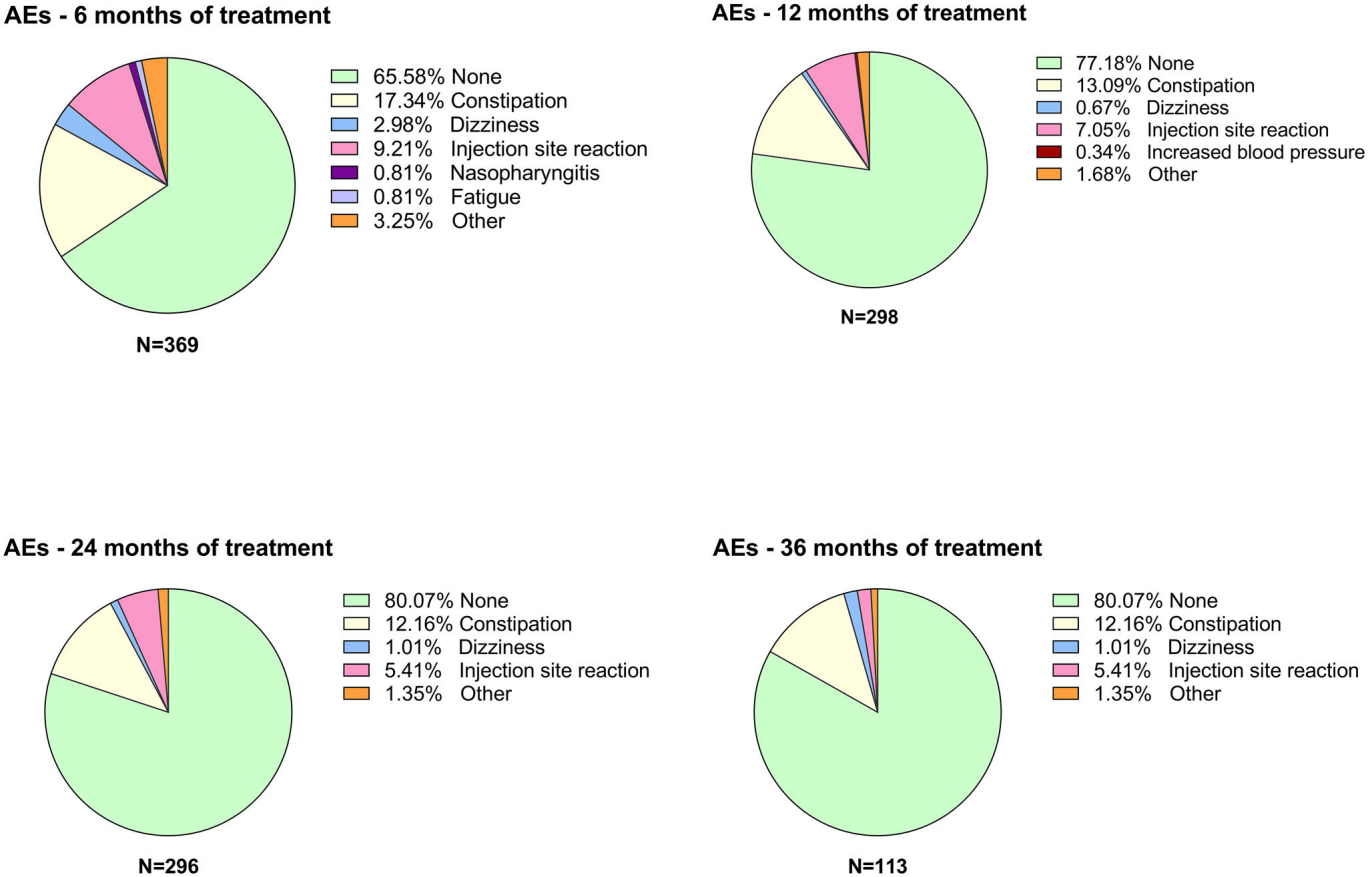
**Representation of the adverse effects experienced by patients after 6, 12, 24, and 36 months of treatment**. Other adverse effects included asthenia, flu‐like symptoms, and Raynaud's phenomenon. The number of cases of each AE is represented, so different AEs of the same patient have been categorized as different cases. Abbreviation: AEs, adverse effects.

Concerning long‐term safety, the therapy was overall well‐tolerated. Up to 80% of patients did not report any adverse events (AEs). Constipation was the most frequently reported AE across all treatment periods, affecting approximately 17% of subjects at 24 months and 13% thereafter. Other infrequently reported AEs included dizziness, local reaction, nasopharyngitis, and fatigue (Figure 3).

**FIGURE 4:**
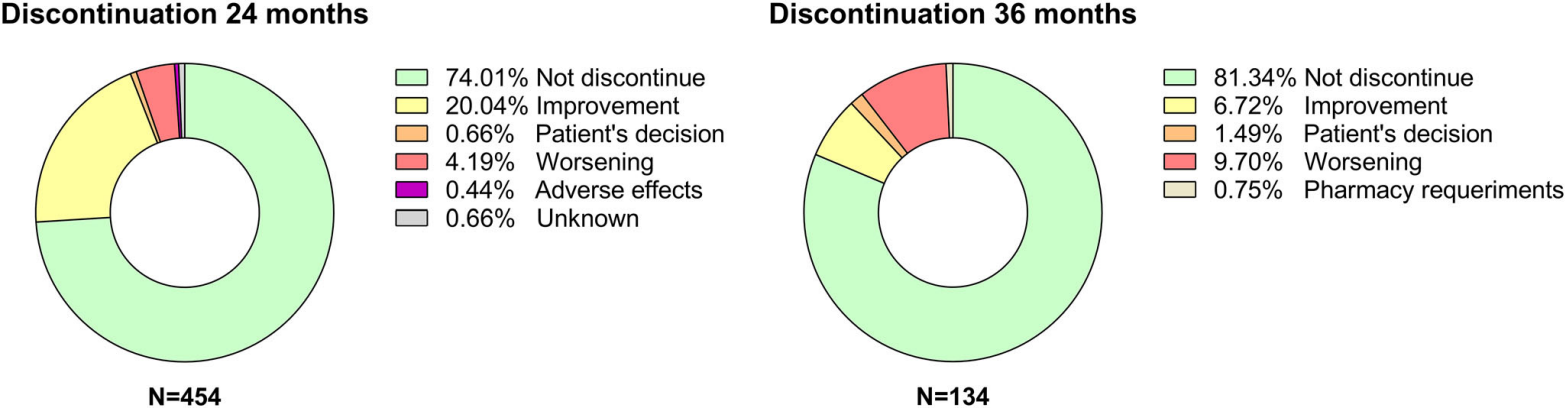
Diagram of reasons for treatment discontinuation after 24 and 36 months of follow‐up.

AEs led to the discontinuation of anti‐CGRP mAb in only two patients (1.7%), both due to local injection site reactions (Figure 4).

It is worth noting that the most common reason for treatment discontinuation after 24 months of anti‐CGRP mAb therapy was clinical improvement (91/454, 20%). Furthermore, loss of effectiveness was observed in only 4.2% of patients after 2 years of treatment. The remaining patients continued with treatment until the study date (Figure 4).

### 3.3 Patient‐Reported Outcomes (PROs)

**FIGURE 5:**
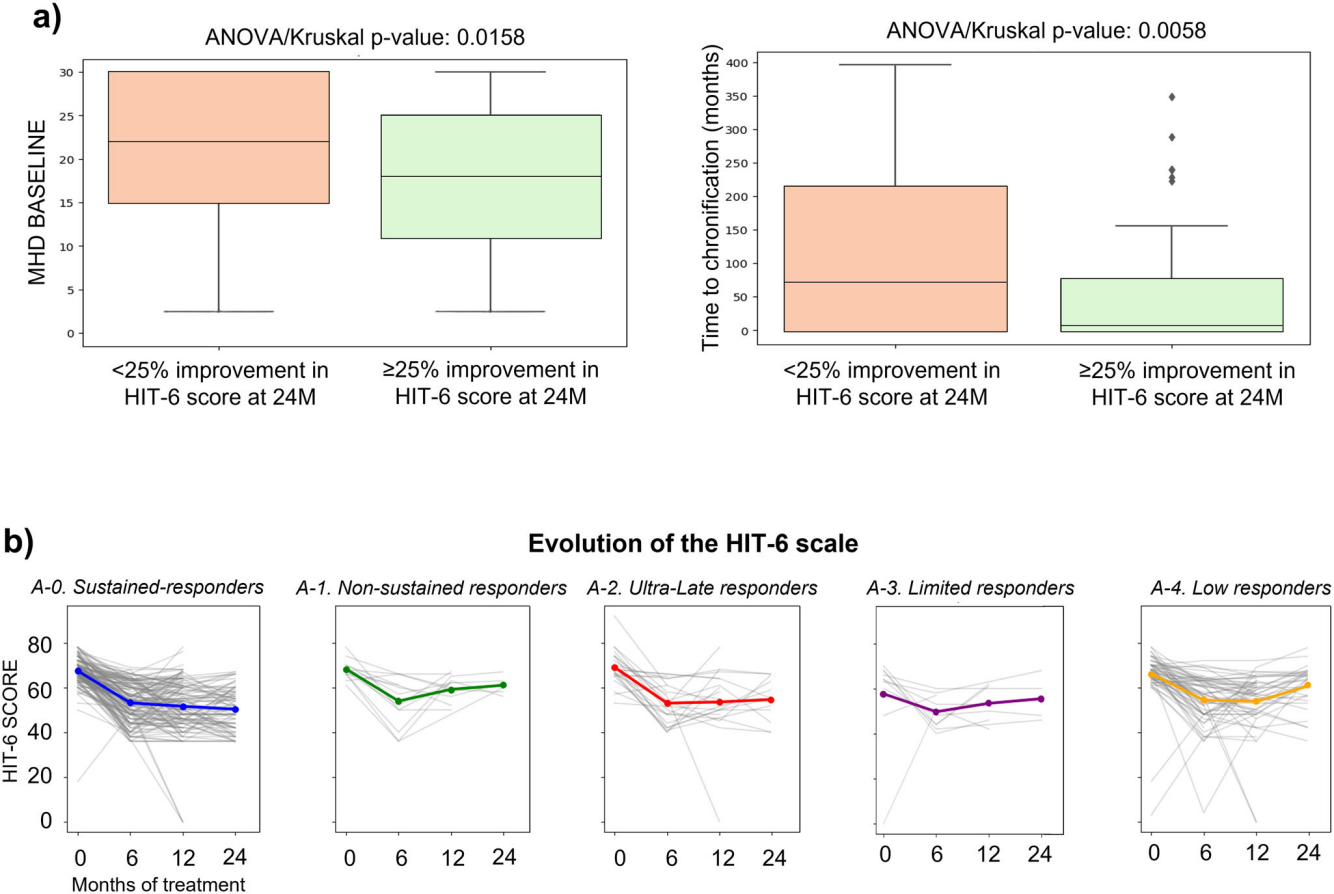
**HIT‐6 scale as a prognostic measure. (a)** Comparison between patients who did not achieve at least a 25% improvement in their HIT‐6 score (orange) and those who did (green) after 24 months of treatment. Box plots represent the median and interquartile range (IQR), with individual data points shown as dots. Error bars represent minimum and maximum values. **(** **b)** Longitudinal evolution of HIT‐6 scores over the course of treatment (0, 6, 12, and 24 months), across patient subgroups defined by their pattern of response. Abbreviations: HIT‐6, Headache Impact Test; MHD, monthly headache days; MMD, monthly migraine days.

At 24 months, patients who achieved a ≥25% improvement in HIT‐6 score exhibited significantly fewer monthly headache days (MHD) at baseline (p = 0.0158) and a shorter time of chronification (p = 0.0058) compared to those who did not show improvement (Figure 5a). These findings suggest that a lower baseline headache burden and a shorter chronification time may be associated with a greater likelihood of responding favorably to treatment over the long term. This should be interpreted as a baseline patient characteristic associated with response, rather than a treatment‐induced change in chronification time.

Among these patients who achieved a HIT‐6 improvement at 24 months, 35% had already demonstrated improvement by 6 months, particularly those with lower baseline MHD and monthly migraine days (MMD). The remaining 65% reached HIT‐6 improvement between months 12 and 24, indicating a delayed but ultimately favorable treatment response.

When analyzing treatment response trajectories (Figure 5b), all patient subgroups exhibited a significant reduction in HIT‐6 scores during the first 6 months of treatment, indicating an improvement in headache‐related impact regardless of response rates based on MHD. This improvement was sustained throughout the second year in patients with a >50% response during this period (A‐0 and A‐2), whereas those with lower response rates (A‐1, A‐3, and A‐4) showed increasing and fluctuating HIT‐6 scores.

### 3.4 Multivariate Analysis

#### 3.4.1 Differential Distribution of MHD and MMD at 24 Months

The multivariate analysis demonstrated that most patients were classified as Sustained responders (A‐0), representing 66.6% when assessed by MHD and 75.1% by MMD. Non‐sustained responders (A‐1) accounted for 4.9% and 7.8%; Ultra‐late responders (A‐2) for 7.3% and 6.4%; Limited responders (A‐3) for 4.7% and 3.8%; and Low responders (A‐4) for 16.6% and 7%, respectively.

**FIGURE 6:**
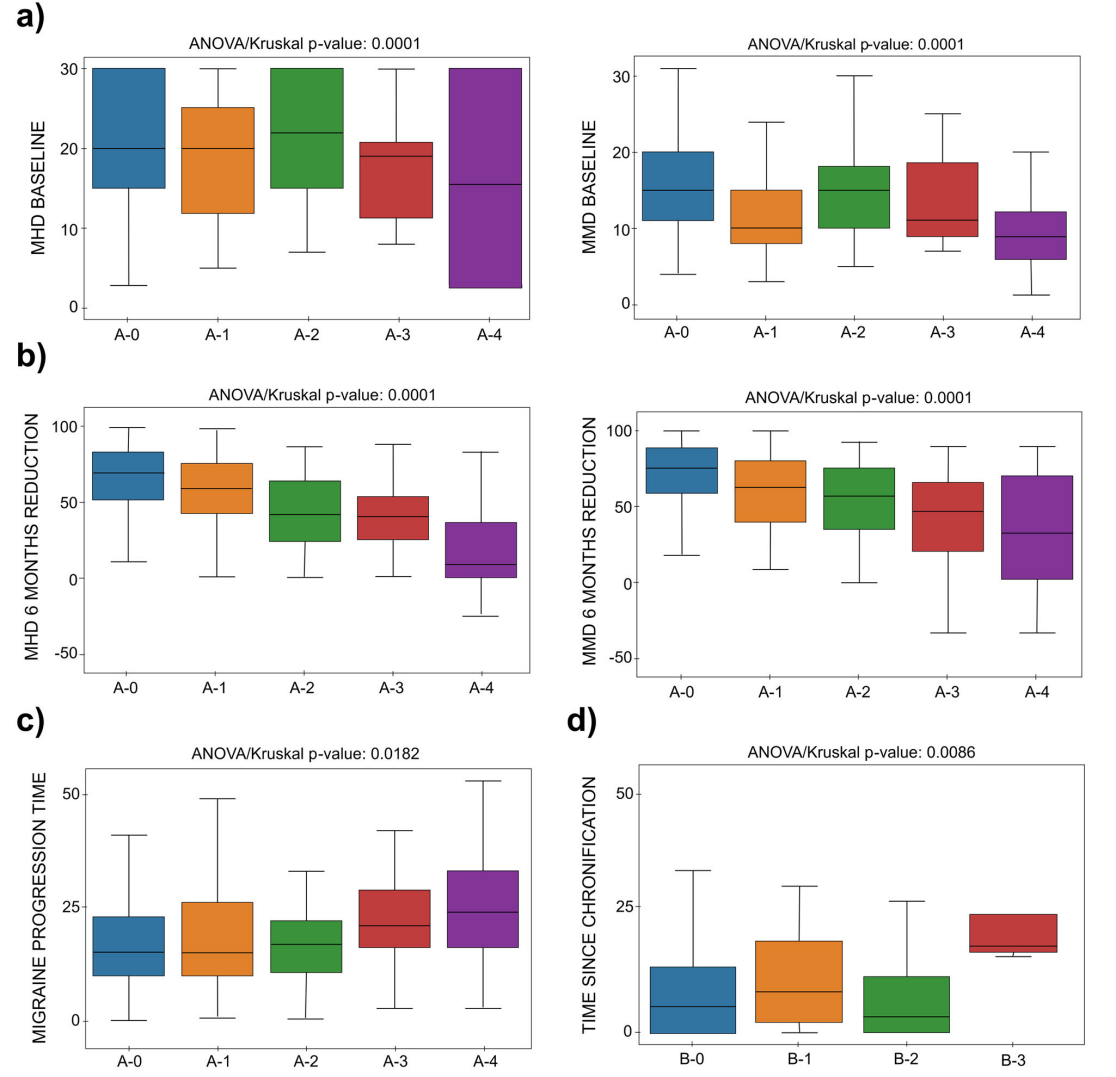
**Clinical differences between patient subgroups stratified by treatment response. (a)** Baseline monthly headache days (MHD) and monthly migraine days (MMD) across response groups at 24 months (A‐0 to A‐4), showing higher baseline values in sustained (A‐0) and ultra‐late responders (A‐2) compared with low responders (A‐4), **(** **b)** Absolute MHD and MMD at 6 months and their reduction from baseline (A‐0 to A‐4), with greater early improvement in sustained responders (A‐0) and significant differences between late responders (A‐2) and low responders (A‐4), **(** **c)** Migraine disease duration at 24 months (A‐0 to A‐4), shorter in sustained (A‐0) and ultra‐late responders (A‐2) than in low responders (A‐4), and **(** **d)** Duration of migraine chronification at 36 months (B‐0 to B‐3), shorter in sustained (B‐0) and ultra‐delayed responders (B‐2) compared with late‐sustained (B‐1) and non‐responders (B‐3). Box plots represent the median and interquartile range (IQR), with individual data points shown as dots. Error bars represent minimum and maximum values.

Several variables showed significant differences in MHD and MMD at 24 months of treatment across the patient subgroups:

- Baseline headache and migraine days were higher in Sustained (A‐0) and Ultra‐late responders (A‐2) than in the Low responders (A‐4), in relation to MHD and MMD, respectively (Figure 6a). Accordingly, the potential for improvement from baseline was larger in patients with more severe baseline conditions.
- At 6 months, reductions from baseline in MHD and MMD were greater in Sustained (A‐0) and Ultra‐late responders (A‐2) compared with Low responders (A‐4) (Figure 6b). Differences were not evident when considering absolute values alone, indicating that absolute headache or migraine days at a single time point may not adequately distinguish response profiles, whereas combining absolute values with the degree of reduction from baseline provides a more accurate differentiation of treatment trajectories.
- Disease duration was significantly shorter in Sustained (A‐0) and Ultra‐late responders (A‐2) groups compared to Low responders group (A‐4) (Figure 6c).

#### 3.4.2 Long‐Term Treatment Response: Comparison Between 36 and 24 Months

In addition, we assessed the evolution of treatment response at 36 months compared to 24 months. Among patients, ultra‐sustained responders (B‐0) comprised 63.6% assessed by MHD and 65.9% by MMD. Late‐sustained responders (B‐1) accounted for 11.4% (MHD) and 13.2% (MMD), while ultra‐delayed responders (B‐2) represented 8.3% (MHD) and 9.3% (MMD). Maintained‐limited responders (B‐3) comprised 16.7% (MHD) and 11.6% (MMD).

Patients who achieved or maintained a ≥50% response at 36 months (B‐0 and B‐2) had a significantly shorter migraine chronification than maintained‐limited responders (B‐3) (Figure 6d).

### 3.5 Evaluation of Predictive Models for Treatment Response

Based on baseline clinical data (e.g., attack frequency, disease duration, and prior treatment failures), it was not possible to develop reliable predictive models of long‐term maintenance of therapeutic response (regardless of response rate, 0%, 30%, 50%, or 75%).

## 4 Discussion

This study represents, to date, the largest real‐world longitudinal cohort of migraine patients treated with calcitonin gene–related peptide (CGRP) mAbs for at least 2 years (*n* = 454). In the context of studies with longer follow‐up durations (≥3 years), our series ranks among the largest reported, together with that of Barbanti et al. (2025). These findings provide valuable evidence on the long‐term effectiveness and tolerability of anti‐CGRP mAbs in routine clinical practice and support their continued use in some patients.

The treatment benefits observed in our cohort during the first 24 months are consistent with those reported in previous clinical trials and real‐world studies (Orlando et al. 2024), confirming the sustained effectiveness of anti‐CGRP mAbs beyond 18 months (Sacco et al. 2022). Moreover, the degree of improvement at 24 months was comparable to that reported in the open‐label extension phases of pivotal randomized trials (Raffaelli et al. 2023), thereby strengthening the external validity of our findings.

Tolerability remained favorable throughout the follow‐up period. Adverse events reported at 24 and 36 months were comparable to those documented during the early treatment phase and in other real‐world studies. The most frequent events were constipation, dizziness, and injection‐site reactions, all mild in intensity. Importantly, no moderate or severe cases were identified, and none fulfilled criteria for serious adverse events such as hospitalization or life‐threatening risk. Only two patients (0.4%) discontinued therapy at 24 months due to mild injection‐site reactions, and no patients discontinued at 36 months due to AEs. These findings reinforce the long‐term safety profile of anti‐CGRP therapies.

In contrast, treatment discontinuation was mainly driven by clinical improvement rather than adverse events. After the 24‐month visit, 20% of the baseline cohort had discontinued therapy, mostly due to marked reductions in migraine frequency and impact. Among the 135 patients who reached the 36‐month visit, 6.7% discontinued during the fourth year. These patterns likely reflect institutional protocols recommending anti‐CGRP discontinuation after one year of therapy. Nevertheless, adherence remained high: 74% of the baseline cohort were still on treatment at 24 months, and 81% of those who reached 36 months continued therapy.

Patient‐reported outcomes (HIT‐6) (Colorado‐Martín et al. 2025) showed that patients with clinically meaningful improvements at 24 months had shorter disease duration and fewer baseline MHD, suggesting earlier therapy may prevent chronic migraine‐related disability. Only 35% improved within the first six months, indicating a delayed‐responder subgroup whose quality of life improved after one year of continuous treatment. This delayed progression likely reflects both time to achieve sustained migraine reduction and gradual restoration of daily function and social engagement. Across subgroups stratified by MHD response, all showed HIT‐6 score reductions within six months, suggesting limited predictive value for long‐term response durability.

Our multivariate analysis further supports the existence of the so‐called Ultra‐late responder subgroup (A‐2), defined as patients achieving a good response (>50% reduction) only after the first year of anti‐CGRP therapy. This subgroup represented a clinically meaningful proportion of patients (6.4%–7.3%). This finding aligns with previous evidence suggesting that therapeutic benefits may emerge later than typically expected. For example, Barbanti et al. (2024), in a prospective observational study, reported that many patients initially classified as non‐responders at six months subsequently improved, with only 8.7% remaining non‐responders at 12 months.

In line with these observations, our analysis between 24 and 36 months revealed distinct trajectories of treatment response. Most patients (B‐0, 65%) maintained a sustained ≥50% response, confirming the long‐term durability of anti‐CGRP mAbs in clinical practice. A minority of patients (B‐1, 12%) experienced diminished benefit after year two, reflecting the “loss of effectiveness” phenomenon reported in real‐world studies, while 9% of patients (B‐2) achieved a late response only at 36 months, supporting therapy beyond 12–18 months in some cases. Finally, persistent non‐responders (B‐3, 15%), likely reflecting longer disease duration and greater migraine chronification, may limit the potential benefits of anti‐CGRP therapies. These findings align with Barbanti et al. (2025), who reported rising ≥50% responder rates over time (25% at 1 year, 53.8% at 2, and 77.8% at 3) and collectively underscore the importance of sustained therapy to maximize long‐term effectiveness.

Within our cohort, patients with chronic migraine and longer disease duration were less likely to sustain responses beyond 24 months, echoing findings from the largest available real‐world study (*N* > 5000) (Caronna et al. 2024), in which higher baseline frequency and disability predicted worse outcomes. This highlights the potential benefit of initiating preventive therapy before the disease becomes highly disabling. However, the literature remains somewhat inconsistent; Ornello et al. (2025), for example, found no significant association between disease duration and treatment response, suggesting that additional factors such as comorbidities and prior treatment history may also modulate long‐term outcomes.

Beyond treatment effectiveness and tolerability, a noteworthy finding of our study is that, despite the large cohort size and extensive clinical characterization of patients, we were unable to develop reliable predictive models for long‐term sustained response. This finding underscores the complexity of migraine pathophysiology and its clinical heterogeneity, which likely exceeds the predictive capacity of conventional baseline variables. Consequently, the identification of clinical, genetic, or neuroimaging biomarkers, as well as the application of artificial intelligence–based algorithms integrating multidimensional data, may be crucial in future research to improve patient stratification and selection for anti‐CGRP mAbs.

To conclude, the majority of patients in our study belonged to the group of excellent Sustained responders (A‐0), defined as maintaining a ≥50% reduction at both 12 and 24 months, with 66% meeting this criterion for monthly headache days (MHD) and 75% for monthly migraine days (MMD). Although the remaining subgroups were smaller, statistically significant differences were observed between distinct response patterns to anti‐CGRP therapy. The presence of patients achieving optimal responses only at later stages (24 and 36 months) underscores the importance of considering disease duration, comorbidities, and baseline clinical status to ensure that each patient derives the maximum benefit from treatment. Overall, our findings emphasize the need for personalized treatment strategies, the value of extending therapy beyond conventional discontinuation points, and the potential of anti‐CGRP mAbs as a long‐term preventive option in some migraine patients.

The main limitation of this study is its retrospective and multicenter design, which may have introduced variability in data collection, use of assessment scales, and follow‐up criteria, particularly beyond the third year of treatment. In many cases, treatment discontinuation was determined by institutional protocols rather than individualized clinical decisions. While this added some heterogeneity, it also enhances the external validity of the findings by reflecting real‐world clinical practice. In addition, the multicenter design represents a key strength of the study, as it increases the generalizability of the results by including a large and clinically diverse population across different headache units with real‐world variability in treatment practices. Another limitation is that, because the study specifically focused on patients who maintained anti‐CGRP monoclonal antibody treatment for at least two years, a survivorship bias may have been introduced, potentially leading to higher long‐term effectiveness estimates compared with unselected cohorts including early treatment discontinuation. However, this design was intended to evaluate the persistence and durability of treatment response and safety among patients with prolonged real‐world exposure. These findings should therefore be interpreted within the context of a long‐term treated population. Sex‐stratified analyses were not performed; however, given the higher prevalence of migraine in women compared with men (approximately 20% vs. 10%), the study population was predominantly female (91%), which limits the assessment of potential sex‐related differences. Moreover, no drug‐specific subgroup analyses were performed, as differences in the temporal availability of anti‐CGRP mAbs and their initial implementation in clinical practice resulted in patient groups with distinct disease histories and cumulative treatment exposure, limiting comparability between anti‐CGRP mAbs. Furthermore, no formal sample size calculation or statistical power analysis was performed due to the retrospective design of the study, which should be considered when interpreting the results. Nonetheless, this design provides unique insight into the long‐term course of patients with sustained benefit and tolerability, offering clinically relevant information that is rarely available in real‐world settings.

## 5 Conclusions

The ESTELA study provides robust evidence on the long‐term effectiveness and safety of anti‐CGRP mAbs in migraine prevention. Sustained reductions in monthly headache and migraine days were observed beyond 2 years of treatment, accompanied by a favorable safety profile and no emergence of new adverse events. The marked decrease in medication overuse further emphasizes their therapeutic value in clinical practice.

Our findings suggest that prolonged treatment may be particularly beneficial in patients with long‐standing chronic migraine and call into question current recommendations for discontinuation after 12–18 months. While interpretation should consider the retrospective and multicenter design, these results provide clinically relevant insights to refine treatment protocols. Prospective, controlled studies are warranted to confirm these observations and define the optimal duration of therapy.

## Author Contributions

**Alba Somovilla**: conceptualization, methodology, investigation, data curation, formal analysis, validation, visualization, writing – original draft. Iris Fernández‐Lázaro: conceptualization, methodology, investigation, data curation, formal analysis, validation, visualization, writing – original draft. Josué Pagán: software, resources, writing – review and editing. **Daniel Saiz**: software, resources, writing – review and editing. **Javier Díaz‐De‐Terán**: resources, writing – review and editing. **Leonardo Portocarrero Sánchez**: resources, writing – review and editing. **Germán Latorre**: resources, writing – review and editing. **Carlos Calle De Miguel**: resources, writing – review and editing. Nuria González‐García: resources, writing – review and editing. **María‐Luz Cuadrado**: resources, writing – review and editing. **Jesús Porta‐Etessam**: resources, writing – review and editing. Javier Casas‐Limón: resources, writing – review and editing. David Garcia‐Azorin: resources, writing – review and editing. **Ángel Guerrero‐Peral**: resources, writing – review and editing. Yésica González‐Osorio: resources, writing – review and editing. **Alicia Gonzalez‐Martinez**: resources, writing – review and editing. Guillermo Martín Ávila: resources, writing – review and editing. Rodrigo Terrero Carpio: resources, writing – review and editing. **Jaime Rodríguez‐Vico**: resources, writing – review and editing. Alex Jaimes: resources, writing – review and editing. **Andrea Gómez García**: resources, writing – review and editing. Cristina Trevino‐Peinado: resources, writing – review and editing. Margarita Sanchez‐Del‐Rio: resources, writing – review and editing. **Alberto Lozano Ros**: resources, writing – review and editing. Antonio Sánchez‐Soblechero: resources, writing – review and editing. Sarai Urtiaga Valle: resources, writing – review and editing. Marta González‐Salaices: resources, writing – review and editing. Elena Riva: resources, writing – review and editing. **Ana Gago‐Veiga**: methodology, conceptualization, investigation, funding acquisition, project administration, supervision, resources, writing – review and editing.

## Funding

This work was supported by the Instituto de Salud Carlos III (ISCIII) and the European Regional Development Fund (ERDF), through IMPaCT project PMP22/00158, and co‐funded by the European Union through the Recovery, Transformation and Resilience Plan—Next Generation EU.

## Authorship

All named authors meet the International Committee of Medical Journal Editors (ICMJE) criteria for authorship for this article, take responsibility for the integrity of the work, and have given their approval for this version to be published.

## Prior Presentation

This study was presented as an oral communication and as part of “Highlighted Communications” at the LXXVI Annual Meeting of the Spanish Society of Neurology, held in Valencia from 19 to November 23, 2024; and at the XXII Annual Meeting of the Madrid Association of Neurology (AMN), held in Madrid on 17–October 18, 2024.

## Ethics Statement

The study was approved by the Ethics Committee of the La Princesa University Hospital (Approval number: 4484) and was conducted in accordance with the ethical principles outlined in the Declaration of Helsinki.

## Conflicts of Interest

Díaz‐De‐Terán J has received speaker honoraria and/or served as a clinical advisor for Novartis, Lilly, Organon, TEVA, Exeltis, Chiesi, Abbvie, Pfizer, Dr. Reddy's, and Lundbeck. Calle de Miguel C has received honoraria from TEVA, Allergan‐Abbvie and Lundbeck. Cuadrado M‐L has received honoraria as a consultant or lecturer for Novartis, Lundbeck and Teva. Porta‐Etessam J has received honoraria from Novartis, Lilly, TEVA, Organon, Allergan‐Abbvie and Lundbeck. Casas‐Limón J has received honoraria as a consultant or speaker for: AbbVie, Almirall, Chiesi, Eli Lilly, Lundbeck, Novartis, Organon, Pfizer and Teva. Garcia‐Azorin D has received honoraria for AbbVie/Allergan, Eli Lilly, Teva, Lundbeck, and Novartis and has participated in clinical trials as the principal investigator for Pfizer, BioHaven, and Lundbeck. Guerrero‐Peral A.L has participated in advisory and speaker boards for Abbvie, Dr. Reddy's, Eli Lilly, Lundbeck, Novartis, Organon, Pfizer and Teva. Gonzalez‐Martinez A has received education funding from Lilly, Novartis, Roche, TEVA, Abbvie‐Allergan, & Daichi and speaker honoraria from TEVA. Martín Ávila G has received honoraria for Lundbeck y Organon. Rodríguez‐Vico J has received honoraria as a consultant and speaker for: AbbVie‐Allergan, Chiesi, Exeltis, Novartis, Eli Lilly, Organon, Grunenthal, Dr. Reddys and Teva. Jaimes A has received speaker honoraria and/or served as a clinical advisor for Lilly, TEVA, Organon, Allergan‐Abbvie and Lundbeck. Trevino‐Peinado C has received honoraria for Organon. Sanchez‐Del‐Rio M has received honoraria for Eli Lily, Lundbeck, Novartis, Teva and Pfizer. Lozano‐Ros A has received honoraria as a consultant and speaker for: Abbvie‐Allergan, Dr. Reddys, Teva, Pfizer and Lundbeck. Sánchez‐Soblechero A has received honoraria from TEVA, Lundbeck, and Organon; and, not related to this research, from Abbvie, Almirall, and Pfizer. Riva E has received honoraria as a consultant and speaker for: Organon and Dr. Reddys. Gago‐Veiga A has received honoraria as a consultant and speaker for: AbbVie‐Allergan, Chiesi, Exeltis, Novartis, Eli Lilly, Organon, Grunenthal, Dr. Reddys and Teva. She is the coordinator and principal investigator of a research IMPaCT project, grant number PMP22/00158. Somovilla A, Fernández‐Lázaro I, Pagán J, Sáiz D, Portocarrero Sánchez L, Latorre G, González‐García N, González‐Osorio Y, Terrero Carpio R, Gómez García A, Urtiaga Valle S, González‐Salaices M have no disclosures to declare.

## Data Availability

The datasets generated during and/or analyzed during the current study are available from the corresponding author on reasonable request.
